# Chikungunya Virus in US Travelers Returning from India, 2006

**DOI:** 10.3201/eid1305.070015

**Published:** 2007-05

**Authors:** Robert S. Lanciotti, Olga L. Kosoy, Janeen J. Laven, Amanda J. Panella, Jason O. Velez, Amy J. Lambert, Grant L. Campbell

**Affiliations:** * Centers for Disease Control and Prevention, Fort Collins, Colorado, USA

**Keywords:** Chikungunya, alphavirus, laboratory diagnosis, RT-PCR, dispatch

## Abstract

Chikungunya virus (CHIKV), a mosquitoborne alphavirus; is endemic in Africa and Asia. In 2005–2006, CHIKV epidemics were reported in islands in the Indian Ocean and in southern India. We present data on laboratory-confirmed CHIKV infections among travelers returning from India to the United States during 2006.

Chikungunya virus (CHIKV) is a mosquito-transmitted virus (genus *Alphavirus*, family *Togaviridae*) usually associated with acute epidemic polyarthralgia. The virus is serologically and genetically most closely related to o’nyong-nyong, Igbo Ora, and, to a lesser extent, Mayaro and Ross River viruses, all of which are associated with acute polyarthralgia (*1*).

CHIKV epidemics have been described in Africa, the Middle East, India, and Southeast Asia, and may have caused epidemics in the Caribbean and in the United States during the early 19th century (*2*). CHIKV epidemics can be explosive with large numbers of human cases and rapid virus dissemination. In the Réunion Island epidemic from April 2005 to June 2006, ≈270,000 cases were reported, representing nearly 40% of the population (*3*). *Aedes aegypti* is the principal vector; however, in recent epidemics in Réunion Island and southern India, *Ae*. *albopictus* has been co-implicated (*4*,*5*). In Africa, CHIKV is maintained in an enzootic cycle involving primates, but in Asia and in recent large epidemics, the human-mosquito cycle predominates, possibly including mechanical transmission (*6*). Symptoms are characterized by acute onset of joint pain, followed by myalgia, fever, and rash with recovery usually within weeks.

Laboratory diagnosis of CHIKV infection is accomplished by serologic methods, virus isolation, and reverse transcription–PCR (RT-PCR). A typical serologic algorithm involves testing acute- and convalescent-phase serum specimens for immunoglobulin M (IgM) and IgG antibody, followed by a plaque reduction neutralization test (PRNT). Virus isolation and RT-PCR are normally used with early acute-phase specimens (before day 5 post-onset) because duration of viremia is typically 2–4 days.

Recent CHIKV outbreaks have been reported in several islands in the Indian Ocean as well as in southern India, where >1 million cases were reported in 2006 (*4*,*7*). CHIKV infections have also been documented in travelers returning from these areas (*3*,*7*). We report confirmed CHIKV infections among 35 travelers returning from overseas travel; 33 were returning from India and 2 from Réunion Island (Table 1).

**Table 1:** Diagnostic test results for 35 travelers infected with chikungunya virus (CHIKV), 2006*

Sample	IgM ELISA†	IgG ELISA†	PRNT‡	Virus isolation (Vero cells)	RT-PCR§	Viremia, PFU/mL¶	Days from onset of illness to collection	State of US residence	Return date, 2006
1	17.7	3.2	640	–	–	NA	0	NJ	10/12
2	1.7	1.7	<10	–	+	10^4.0^	1	CA	Before 11/28
3	1.2	1.1	ND	+	+	10^4.1^	1	IL	9/29
4	1.8	NS	ND	+	+	10^6.8^	2	CA	Before 9/16
5	1.2	0.76	ND	+	+	10^5.1^	2	MA	9/10
6	1.8	1.6	<10	+	+	10^6.0^	3	PA	Before 8/20
7	1.6	1.2	ND	+	+	10^5.3^	3	CA	10/2
8	NS	1.4	ND	–	+	10^3.9^	4	WI	10/9
9	22.0	4.3	5,120	–	–	NA	4	CA	Before 10/6
10	1.1	0.95	<10	–	+	10^4.5^	6	CA	Before 8/13
11	7.4	0.96	40	–	–	NA	7	CA	Before 9/23
12	15.0	0.60	320	–	–	NA	8	CT	Before 7/6
13	26.2	1.2	160	ND	–	NA	8	DC	Before 10/16
14	12.9	5.8	1,80	ND	–	NA	10	CA	Before 9/22
15	38.8	2.3	2,560	ND	ND	NA	19	CT	Before 7/6
16	12.7	1.5	640	ND	–	NA	20	IL	8/23
17	16.9	4.8	640	ND	–	NA	30	IL	6/25
18	8.3	3.7	640	ND	ND	NA	31	HI	Before 8/2
19	6.6	1.5	640	ND	–	NA	31	CA	Before 8/13
20	2.6	6.8	320	ND	–	NA	34	MD	Before 3/20
21	5.0	NS	640	ND	–	NA	34	IL	Before 9/22
22	15.1	4.1	1,280	ND	–	NA	38	CA	Before 10/2
23	4.3	5.9	2,560	ND	–	NA	42	PA	Before 8/20
24	5.6	NS	320	ND	–	NA	43	Unknown	Unknown
25	3.1	3.7	640	ND	–	NA	44	IL	Before 9/12
26	26.6	13.4	5,120	ND	ND	NA	48	SC	6/24
27	30.7	6.6	5,120	ND	–	NA	61	CA	Before 10/26
28	6.1	11.4	2,560	ND	–	NA	62	CT	Before 10/3
29	9.4	16.0	640	ND	–	NA	63	CA	8/23
30	7.5	8.4	1,280	ND	–	NA	71	MN	6/8
31	5.3	5.8	640	ND	–	NA	71	MN	6/8
32	3.7	16.1	320	ND	–	NA	75	LA	Before 3/30
33	9.8	10.1	2,560	ND	–	NA	92	IL	7/9
34	7.5	3.1	160	ND	–	NA	101	PA	Before 10/13
35	24.6	11.9	20,480	ND	–	NA	Unknown	IL	Before 11/08

*IgM, immunoglobulin M; PRNT, plaque reduction neutralization test; RT-PCR, reverse transcription–PCR; NA, not applicable; ND, not done (sampled depleted); NS, nonspecific reaction in ELISA. †Values are patient sample optical densities divided by a negative control optical density; values >3 are positive. ‡Values are 90% plaque reduction neutralization titers. §Real-time, fluorescence-based assay for detecting CHIKV RNA; positive samples had crossing threshold values <37 with both primer sets. ¶Estimated CHIKV PFU/mL by real-time RT-PCR using a standard curve generated with plaque-titrated/calibrated CHIKV standards.

## The Study

Serum samples were received by the Centers for Disease Control and Prevention (Fort Collins, CO, USA) from April 2006 to December 2006 as part of routine diagnostic and reference services available to public health laboratories. A total of 106 serum samples were received from persons returning from regions with epidemics or where CHIKV is endemic (79 from India and the Indian Ocean islands and 27 from Africa) with compatible CHIKV illness and submitted by state public health laboratories. Serum samples were tested for antibodies to several viruses known to occur in the region of travel and residence by IgM capture ELISA and a standard IgG ELISA (*8*,*9*). The 35 CHIKV IgM- and IgG-positive specimens were tested by using a PRNT (90% reduction cutoff) with several related alphaviruses (Sindbis, o’nyong-nyong, and Semliki Forest viruses) to confirm specificity of reactivity (*10*). A ≥4-fold neutralizing titer difference between antibody to CHIKV and antibodies to other alphaviruses indicated a CHIKV-specific antibody response. IgM-positive and PRNT specificity–confirmed specimens were classified as recent CHIKV infections (Table 1).

All serum specimens were tested by a quantitative, real-time, fluorescent probed–based RT-PCR assay for CHIKV RNA. Two primer probe sets were designed in unique regions of the viral genome and reacted specifically with CHIKV RNA and not with related or unrelated viruses (Table 2). Both sets showed an analytical sensitivity <1 PFU, and CHIKV was detected in virus-spiked serum samples at a concentration of 10 PFU/mL (75 μL of serum assayed). Eight serum specimens showed positive results by the real-time assay; all were acute-phase specimens with number of days post-onset of illness reported as <6. Viral titers of these specimens were estimated by quantitative RT-PCR that used CHIKV quantity standards (determined by plaque assay) to generate a standard curve. Titers of 8 specimens ranged from 10^3.9^ PFU/mL to 10^6.8^ PFU/mL.

**Table 2:** Sensitivity and specificity of chikungunya virus (CHIKV) oligonucleotide primers used in real-time reverse transcription–PCR assay

Primer	Genome position*	Sequence (5′→3′)	Sensitivity†	Specificity‡
CHIKV 874	874–894	AAAGGGCAAACTCAGCTTCAC		
CHIKV 961	961–942	GCCTGGGCTCATCGTTATTC	0.3	CHIKV
CHIKV 899-FAM§	899–923	CGCTGTGATACAGTGGTTTCGTGTG		
CHIKV 6856	6856–6879	TCACTCCCTGTTGGACTTGATAGA		
CHIKV 6981	6981–6956	TTGACGAACAGAGTTAGGAACATACC	0.9	CHIKV
CHIKV 6919-FAM	6919–6941	AGGTACGCGCTTCAAGTTCGGCG		

*On the basis of CHIKV prototype strain S27, GenBank accession no. NC_004162. †Absolute no. of PFU detected in triplicate testing. ‡No reactivity was observed with the following viruses: o’nyong nyong, Ross River, Mayaro, Semliki Forest, Sindbis, western equine encephalitis, eastern equine encephalitis, and Venezuelan equine encephalitis subtypes 1AB, 1C, 1D, and 1E. §Primer labeled at the 5′ terminus with 5-FAM and 3′ Black Hole Quencher 1 (Operon Biotechnologies Inc., Huntsville, AL, USA).

All acute-phase specimens (on or before day 8 postonset) were also tested for CHIKV by virus isolation in Vero cells. Isolation was performed by using a recently developed protocol in which cells were grown in glass shell vials and centrifuged to enhance viral infectivity (J.O. Velez, unpub. data). Five serum specimens displayed prominent and characteristic cytopathic effect on day 2 postinfection, and virus was identified as CHIKV by RT-PCR. All virus isolates were obtained from acute-phase specimens that also were positive by RT-PCR. Three serum specimens (samples 2, 8, and 10) showed positive RT-PCR results, but CHIKV was not isolated from these specimens. In these 3 specimens, inability to isolate virus may have been related to viral titers, which were lower than most of the virus isolation–positive samples, or to handling or storage of these samples. All 8 virus-positive specimens (whether positive by RT-PCR, virus isolation, or both) were collected <7 days postonset and were negative for IgM and IgG antibodies to CHIKV. Nearly all of the specimens collected <7 days postonset were positive by 1 of the virus-based tests. The 2 exceptions, samples 1 and 9, were positive for IgM and IgG antibodies to CHIKV and had high PRNT titers. These findings indicate that these samples were not true acute-phase specimens; the true onset or collection date had likely been reported incorrectly.

To identify the strain of CHIKV in these specimens, a 2,122-bp fragment from the structural region of the genome (nucleotide positions 9,648–11,770) was amplified from all 8 virus-positive specimens by RT-PCR and subjected to nucleic acid sequencing with previously described primers (*11*). All 8 sequences showed nucleotide identity >99.7% (GenBank accession nos. EF451142–EF451149). BLAST analysis (www.ncbi.nlm.nih.gov/blast) of the 8 sequences showed that the highest percentage identity was to CHIKV strains recently isolated from travelers returning from Indian Ocean islands (Réunion, Mauritius, and Seychelles). Percentage identity matches between the 8 viruses and Indian Ocean CHIKV strains were >99.5%, with 5 to 8 mismatches occurring randomly. In comparison, percentage identities of the 8 viruses to CHIKV prototype S27 or to a strain previously isolated from India (Nagpur/653496) were 95.1% and 94.4%, respectively.

## Conclusions

The data reported confirm that the widespread CHIKV epidemic in southern India has infected US travelers. CHIKV infections among international travelers are not unexpected; in 2005–2006, ≈800 CHIKV infections were reported in France, primarily in travelers returning from Réunion Island (*3*). The more noteworthy observation of this study with potential public health ramifications is that high levels of infectious virus were detected in returning travelers. Primary vectors for CHIKV are *Ae*. *aegypti* and *Ae*. *albopictus*, which are established in several southeastern coastal states in the United States. Vector competence studies of *Ae*. *aegypti* and *Ae*. *albopictus* strains from the United States, as well as strains from the Caribbean and South America, showed that a titer of ≈10^4^ PFU/mL in monkeys resulted in productive infection with virus dissemination in these mosquitoes, with *Ae*. *albopictus* showing higher infection and dissemination rates (*12*). The level of viremia reported in most of these imported CHIKV infections, >10^4^ PFU/mL, could be sufficient to infect North American vectors, given the appropriate environmental conditions. However, the time of year and place of residence of the returning travelers in this study were not conducive to transmission; only 2 (patients 26 and 32) returned to US regions (South Carolina and Louisiana) known to have populations of *Ae*. *albopictus*.

Nevertheless, returning travelers with high viremia levels, who live in areas with established *Ae*. *aegypti* and *Ae*. *albopictus* populations, could facilitate local transmission in the United States. Clinicians should therefore obtain travel histories from persons with CHIKV-compatible illness and include CHIKV in differential diagnoses when appropriate. Public health laboratories must carefully monitor CHIKV infections of returning travelers and conduct surveillance for CHIKV-infected vectors in high-risk areas to prevent local establishment of a new emerging virus. Diagnostic laboratory personnel involved in virus isolation protocols must be aware of the potential of isolating CHIKV (a biosafety level 3 agent) from patients returning from regions endemic for CHIKV or regions with epidemics and take appropriate safety precautions.
